# Placenta Previa1Three cases given in connection with a paper read before the Wyoming County Medical Association, January 14, 1896.

**Published:** 1896-05

**Authors:** Wm. Stanton

**Affiliations:** Varysburg, N. Y.


### Clinical Report.

### PLACENTA PREVIA.[1]

By WM. STANTON, M.D., Varysburg, N. Y.

CASE I.—*Placenta previa partialis.* Mrs. G. P., aged 20. About thirty-eight weeks advanced in first pregnancy. Was called at 4 A. M., June 13, 1895, to attend her in labor. Found she had had dragging pains all the previous day and night, which had become

1. Three cases given in connection with a paper read before the Wyoming County Medical Association, January 14, 1896.

nearly constant ; no hemorrhage. Not being suspicious of any complication, a simple digital examination revealed a soft, patulous os. Could have easily introduced one or two fingers, but did not, a vertex presentation being evident. I gave her 4 gr. quinine and went home to breakfast. Calling about 7.30 A.M., found her easy, only having occasional light pains. She was about the house and quite comfortable most of the day.

In the evening the pains increased and slight hemorrhage began. Having made no examination since early morning I now passed the index finger through the os, and, to my surprise, came upon the placenta, covering the fetal head. On the right side it was still firmly attached, but on the left it was partly loosened. Carefully passing the finger high up, I could just reach the margin of the placenta. I now gave a full dose of ergot, informed her husband of the condition and by questioning obtained history of two hemorrhages,—one at about the twenty-sixth week, lasting two or three days ; the second, about four weeks later. Being able to reach an edge of the placenta and having a vertex presentation, I determined, if possible, to secure a vertex delivery. Strong pains soon followed, and hemorrhage increasing, I loosened the placentaas far as possible and ruptured the membranes. With the right hand securing dilatation and pushing the placenta to one side, while with the left I used Kristeller's expression, the engagement of the head was soon accomplished, when all hemorrhage ceased. She made but little progress, however, the placenta becoming an obstruction. I therefore administered chloroform and delivered with forceps, immediately removing the placenta and firmly grasping the uterus through the abdominal walls, controlled the hemorrhage. Mother and child both did well.

CASE II.—*Placenta previa centralis.* Mrs. J. A., aged 42. Sixth pregnancy ; at term. Previous gestations normal. No history of hemorrhage. Had suffered greatly during the later months from extreme soreness in the lower part of the abdomen, groins and pelvis ; felt more when standing and rendering locomotion almost impossible.

Was called just before dark, August 8, 1895, four miles in the country. Upon my arrival all pains had ceased for the first time in thirty-six hours. Examination revealed a patulous os, which gave but little resistance to the introduction of the index finger, when I came upon a smooth, boggy substance, feeling exactly like the fetal surface of a normal placenta ; still the membranes had not ruptured and there had been no hemorrhage.

Vertex presentation was evident, the patient quite easy and likely to secure a few hours' rest. I therefore decided to remain and watch developments. After a few hours of occasional pains, a second examination was made and with some difficulty two fingers introduced within the os, when I found the margin of the membrane over the placenta firmly adherent all around at some 4 to 6 centimeters from the mouth. The administration of ergot secured strong and regular pains, and dilatation sufficient to easily allow the introduction of three fingers was secured, with absolutely no hemorrhage. I now determined to loosen the placenta and reach an edge, if possible. Having failed to perform version by external manipulation, the pelvis being fairly roomy and contractions good, I decided to attempt a vertex delivery. Considerable difficulty was met in loosening the edge of the membrane and it was quite painful to the patient. After loosening the placenta as far as possible, I could not reach the edge at any place. I therefore tore through and thus ruptured the membranes. The liquor escaped with a gush, and a strong pain, aided by Kristeller's expression, immediately pressed the head down, sufficient to check hemorrhage. The placenta here, as in Case I., became an obstruction, and delivery was completed in the same manner. After delivery the membranes were found entire, the only opening being through the placenta, which was very large, of medium thickness and had on its maternal surface, almost exactly in the center, a patch of membrane, about 6 centimeters in diameter, similar to that covering the fetal side and very tough. Mother and child both did well.

Case III. Mrs. J. W., aged 32. About seventeen or eighteen weeks advanced in third pregnancy. Previous pregnancies normal.

Was called about 2 A.M., December 4, 1895, and obtained the following history: She had supposed herself pregnant until seven weeks previous, when she began to flow and continued to do so almost constantly until the previous evening, when pains began and flowing increased so much as to cause alarm. I found her flowing badly and having strong pains. A digital examination revealed a pregnant uterus, patulous os and the placenta squarely planted over the os. I was able to almost completely loosen the placenta by sweeping the finger around within the os. Hemorrhage ceased at once and she was soon delivered without any further difficulty, the placenta being expelled first. Recovery was good and uneventful.

---

Tuberculosis and Genital Infection.—Dobroklonsky, (*Med. Standard,*) from careful experiments (Vratch) concludes that tubercular infections may be conveyed through the genital apparatus from man to woman and vice versa, but only when there are present in these organs tubercular foci, a condition rarely met with. This method of infection in comparison to others is but of secondary importance, however, as local tubercular process in the genital apparatus, either in the male or female, may take such a course as not to cause general infection or threaten life, and may even be latent, existing without the knowledge of the patient himself.

